# MyD88 is pivotal for immune recognition of *Citrobacter koseri *and astrocyte activation during CNS infection^†^

**DOI:** 10.1186/1742-2094-8-35

**Published:** 2011-04-16

**Authors:** Shuliang Liu, Tammy Kielian

**Affiliations:** 1 Department of Neurobiology and Developmental Sciences, University of Arkansas for Medical Sciences, Little Rock, AR 72205 USA; 2 Department of Pathology and Microbiology, University of Nebraska Medical Center, Omaha, NE 68198 USA; 3 Division of Neurotoxicology, National Center for Toxicological Research, FDA, Jefferson, AR 72079 USA

**Keywords:** MyD88, Toll-like receptor 4 (TLR4), *C. koseri*, meningitis, brain abscess, astrocyte

## Abstract

*Citrobacter koseri *(*C. koseri*) is a Gram-negative bacterium that can cause a highly aggressive form of neonatal meningitis, which often progresses to establish multi-focal brain abscesses. The roles of Toll-like receptor 4 (TLR4) and its signaling adaptor MyD88 during CNS *C. koseri *infection have not yet been examined, which is important since recent evidence indicates that innate immune responses are tailored towards specific pathogen classes. Here TLR4 WT (C3H/FeJ) and TLR4 mutant (C3H/HeJ) mice as well as MyD88 KO animals were infected intracerebrally with live *C. koseri*, resulting in meningitis and ventriculitis with accompanying brain abscess formation. MyD88 KO mice were exquisitely sensitive to *C. koseri*, demonstrating enhanced mortality rates and significantly elevated bacterial burdens compared to WT animals. Interestingly, although early proinflammatory mediator release (i.e. 12 h) was MyD88-dependent, a role for MyD88-independent signaling was evident at 24 h, revealing a compensatory response to CNS *C. koseri *infection. In contrast, TLR4 did not significantly impact bacterial burdens or proinflammatory mediator production in response to *C. koseri*. Similar findings were obtained with primary astrocytes, where MyD88-dependent pathways were essential for chemokine release in response to intact *C. koseri*, whereas TLR4 was dispensable; implicating the involvement of alternative TLRs since highly enriched astrocytes did not produce IL-1 upon bacterial exposure, which also signals via MyD88. Collectively, these findings demonstrate the importance of MyD88-dependent mechanisms in eliciting maximal proinflammatory responses, astrocyte activation, and bacterial containment during CNS *C. koseri *infection, as well as a late-phase MyD88-independent signaling pathway for cytokine/chemokine production.

## Introduction

*Citrobacter koseri *(formerly known as *C. diversus*) is a Gram-negative bacillus with a predilection for causing meningitis and multi-focal brain abscesses in human neonates [1,2]. In fact, almost one-third of infants and young children infected with *C. koseri *succumb to the disease, and approximately half of those who survive infection experience long-term neurological deficits due to focal or diffuse brain damage [1,2]. Increasing evidence has accumulated demonstrating that innate immune responses are tailored towards specific pathogen classes [3-5]. Specifically, the types of responses elicited by Gram-positive bacteria can differ significantly from those triggered by Gram-negative pathogens. In addition, the majority of studies examining bacterial pathogenesis in the CNS have utilized Gram-positive organisms [6-8], which eliminates the involvement of key Toll-like receptors (TLRs) that may trigger distinct pathways during Gram-negative infections (i.e. TLR4, TLR5). Therefore, it is important to investigate the CNS response to divergent pathogens to identify unique as well as conserved responses, which may facilitate the development of novel treatment strategies that would cross multiple bacterial species.

The innate immune system recognizes multiple pathogen classes via highly conserved molecular motifs, termed pathogen-associated molecular patterns (PAMPs), through a limited set of germ-line encoded receptors known as pattern recognition receptors (PRRs) [9,10]. Toll-like receptors (TLRs) are a family of PRRs responsible for sensing numerous PAMPs of bacterial, viral, and fungal species [9]. For example, lipoproteins and LPS contained in the outer cell wall of Gram-negative bacteria are agonists for TLR2 and TLR4, respectively; flagellin, which is the main component of bacterial flagella, engages TLR5; and bacterial DNA containing unmethylated CpG motifs binds to TLR9. Since *Citrobacter *are Gram-negative bacilli and possess an outer cell wall rich in LPS, it was anticipated that TLR4-mediated signaling would be important in eliciting host inflammatory responses. However, LPS is not the only *Citrobacter-*derived PAMP that could be recognized by the host. Other candidates include, but are not limited to, lipoproteins, flagellin, and bacterial DNA that are recognized by TLR2, TLR5, and TLR9, respectively. Therefore, all of these TLRs could conceivably contribute to activation of the host inflammatory response during CNS *C. koseri *infection and since they all utilize the common adaptor molecule myeloid differentiation factor 88 (MyD88) [11,12], we also examined responses to bacterial challenge in MyD88 KO mice.

Although their primary function is to provide support for maintaining CNS homeostasis, astrocytes can also participate in innate immune responses and serve as a major source of chemokines [13,14]. Astrocytes express TLRs and can clearly contribute to innate immune processes in the CNS [13,15]. Although astrocytes are strategically positioned at the blood-brain barrier, a site where *C. koseri *must traverse to colonize the CNS parenchyma during infection, to date no studies have examined whether astrocytes are capable of recognizing this pathogen and the downstream consequences elicited. In addition, since astrocytes are the most numerous cell type in the CNS parenchyma these cells likely play a major role in dictating the course of *C. koseri *ventriculitis, meningitis, and brain abscess formation.

Following TLR engagement, a number of diverse signaling pathways can be triggered, the nature of which depends on the specific PAMP [9,16]. Most TLR-mediated signaling pathways converge to utilize the common intracellular adaptor molecule MyD88, with the exception of TLR3. However, in addition to utilizing MyD88, TLR4-mediated signaling can also occur through alternative adapter molecules, namely TIR-domain-containing adaptor protein-inducing IFN-β (TRIF) and TRIF-related adaptor molecule (TRAM) [9,16]. The MyD88-dependent pathway is responsible for early-phase NF-κB and MAPK activation, which induces proinflammatory cytokine/chemokine expression. In contrast, the MyD88-independent, TRIF-dependent pathway activates interferon regulatory factor 3 (IRF3), which is required for the expression of IFN-inducible genes. Furthermore, this pathway mediates late-phase NF-κB as well as MAPK activation, also contributing to inflammatory responses [17]. Since *C. koseri *is a Gram-negative pathogen with abundant LPS in its outer cell wall, it was important to consider the possible contribution of MyD88-independent pathways for eliciting inflammatory gene expression during CNS infection.

To assess the functional importance of TLR4 and MyD88 in *C. koseri-*induced parenchymal infection, TLR4 WT (C3H/FeJ) and TLR4 mutant (C3H/HeJ) mice as well as MyD88 KO animals were infected intracerebrally with live *C. koseri*. MyD88 KO mice were exquisitely sensitive to *C. koseri *and succumbed to infection within 24-36 h following bacterial exposure. The enhanced mortality rate of MyD88 KO mice was typified by significantly elevated bacterial burdens and attenuated cytokine/chemokine expression and immune cell influx early after infection (i.e. 12 h). Interestingly, MyD88-independent pathways were triggered with a delayed kinetics (i.e. 24 h), likely representing a compensatory mechanism in an attempt to contain infection. In contrast to MyD88, TLR4 had minimal impact on bacterial burdens or proinflammatory mediator production. Similar findings were obtained with astrocytes, where MyD88-dependent signaling was essential for chemokine production by *C. koseri *treated cells, whereas TLR4 was dispensable. Collectively, these findings demonstrate the importance of MyD88-dependent mechanisms in eliciting maximal proinflammatory responses and bacterial containment during CNS *C. koseri *infection, as well as a late-phase MyD88-independent signaling pathway for cytokine/chemokine production.

## Materials and methods

### Mouse strains

MyD88 KO mice (kindly provided by Dr. Shizuo Akira, Osaka University, Osaka, Japan) have been backcrossed with C57BL/6 mice for over 10 generations and age- and sex-matched C57BL/6 mice were purchased from the National Cancer Institute (NCI-Frederick, Frederick, MD) as WT controls. C3H/FeJ and C3H/HeJ mice were purchased from the Jackson Laboratory (Bar Harbor, ME). The C3H/HeJ strain carries a spontaneous mutation in the cytoplasmic domain of TLR4 making it unable to transduce a signal in response to LPS (hereafter referred to as TLR4 mutant) [18,19] and C3H/FeJ mice were used as WT controls.

### Ethical approval

The animal use protocols were approved by the Institutional Animal Care and Use Committees at the University of Nebraska Medical Center and University of Arkansas for Medical Sciences, and are in accord with NIH guidelines for the use of rodents.

### Citrobacter koseri isolate

*C. koseri *strain 4036 was originally isolated from the cerebrospinal fluid of an infant with meningitis and multiple brain abscesses [20] and was kindly provided by Dr. J. G. Vallejo (Baylor College of Medicine, Houston, TX). Based on antibiotic sensitivity profiles (performed by the Clinical Microbiology Laboratory at Arkansas Children's Hospital, Little Rock, AR), the strain was propagated in the presence of ampicillin (20 μg/ml) in brain-heart infusion broth with constant agitation (250 rpm) and recovered at mid-log-phase (12-18 h incubation) by washing twice with ice-cold PBS. To prepare live *C. koseri *stocks, freshly recovered bacteria were resuspended in PBS containing 10% DMSO and 5% bovine serum albumin (Sigma-Aldrich, St. Louis, MO) and aliquots stored at -80°C. Immediately before infection, aliquots were washed twice with ice-cold PBS to remove residual DMSO, serially diluted 10-fold in PBS, and plated on blood agar plates to determine infectious titers.

### Generation of *C. koseri*-induced meningitis and brain abscess

Age- and gender-matched TLR4 WT and TLR4 mutant, or C57BL/6 WT and MyD88 KO mice (4-5 wks old) were used for *in vivo *studies to investigate *C. koseri*-induced meningitis and brain abscess pathogenesis. Briefly, live bacteria were directly inoculated into the brain parenchyma by stereotactic injection as previously described [21]. *C. koseri *was resuspended in PBS rather than encapsulated in agarose beads, the latter of which represents the standard method for inducing experimental brain abscesses [22]. The rationale for this approach is that the meninges are punctured during intracerebral injections and the introduction of bacteria in an aqueous suspension allows for pathogen reflux into the subarachnoid space via the needle tract, leading to meningitis formation. This procedure recapitulates clinical disease since many infants infected with *C. koseri *develop meningitis and ventriculitis that is often complicated by abscess formation [2]. Since pilot studies indicated that MyD88 KO animals were extremely sensitive to *C. koseri*, succumbing to infection within the first 24-36 h following bacterial exposure, MyD88 KO and WT mice were euthanized at either 12 or 24 h post-infection to eliminate potential bias from only examining animals that had survived the infection (i.e. "survival bias").

### Preparation of brain homogenates for quantitation of bacterial titers and proinflammatory mediator production

Mice were sacrificed with an overdose of inhaled isoflurane and perfused transcardially with ice cold PBS. Both the infected and contralateral hemispheres were collected, weighed, and homogenized in 500 μl of homogenization buffer [1X PBS supplemented with a protease inhibitor cocktail tablet (Roche, Indianapolis, IN) and RNase inhibitor (Promega, Madison, WI)] on ice. To quantitate the numbers of viable bacteria, serial 10-fold dilutions of brain tissue homogenates were plated onto blood agar. Titers were calculated by enumerating colony growth and are expressed as colony forming units (CFU) per gram wet tissue weight. Supernatants were collected from brain homogenates following centrifugation at 14,000 rpm for 20 min at 4°C for downstream analyses of cytokine and chemokine expression by Milliplex multi-analyte bead arrays. Protein concentrations of brain homogenates were determined using a colorimetric Bio-Rad DC Protein Assay kit as specified by the manufacturer (Bio-Rad, Hercules, CA).

### Milliplex multi-analyte bead array

To compare proinflammatory mediator expression profiles between the various mutant and WT mice, a custom-designed mouse cytokine/chemokine microbead array was utilized according to the manufacturer's instructions (Milliplex; Millipore, Billerica, MA). This microbead array allows for the simultaneous detection of 19 individual inflammatory molecules in a single 75 μl brain homogenate including IL-1α, IL-1β, TNF-α, IFN-γ, IL-6, IL-9, IL-10, IL-12p70, IL-12p40, IL-15, IL-17, CXCL1/keratinocyte chemoattractant (KC), CXCL2/macrophage inflammatory protein-2 (MIP-2), CXCL9/monokine induced by IFN-γ (MIG), CXCL10/IFN-γ-induced protein 10 (IP-10), CCL2/monocyte chemoattractant protein-1 (MCP-1), CCL3/MIP-1α, CCL4/MIP-1β, and CCL5/regulated upon activation T cell expressed and secreted (RANTES). Results were analyzed using a Bio-Plex workstation (Bio-Rad) and adjusted based on the amount of total protein extracted from brain tissue homogenates for normalization.

### Quantitation of cellular infiltrates in *C. koseri *infected brain tissues by FACS

To determine whether MyD88 loss affected neutrophil or macrophage influx and/or microglial percentages following *C. koseri *infection, cells were quantitated by FACS analysis as previously described [6,23]. Briefly, mice were perfused with PBS to eliminate leukocytes from the vasculature and both hemispheres were collected to recover infection-associated cells. Brain tissues were minced in HBSS supplemented with 10% FBS and filtered through a 70 μm nylon mesh strainer. The resulting tissue suspensions were digested for 30 min at 37°C in HBSS supplemented with collagenase type I (Sigma-Aldrich) and DNase I (Invitrogen, San Diego, CA; 2 mg/ml and 28 U/ml final concentrations, respectively) to obtain a single-cell suspension. Following enzyme neutralization, cells were layered onto a discontinuous Percoll gradient (1.03-1.088 g/ml), whereupon myelin debris was carefully aspirated and the cell interface collected. Following extensive washes and incubation in Fc Block™ (BD Biosciences, San Diego, CA) to minimize non-specific antibody binding to Fc receptors, cells were stained with directly conjugated Abs (Ly-6G-PE, F4/80-Alexa 488, and CD45-APC, all from BD Biosciences) to identify neutrophils (Ly-6G^+^, F4/80^-^, and CD45^high^), macrophages (Ly-6G^-^, F4/80^+^, and CD45^high^), and microglia (Ly-6G^-^, F4/80^+^, and CD45^low-intermediate^). The vital stain 7-AAD (eBiosciences, San Diego, CA) was included to discriminate viable from dead cells. Cells were analyzed using a BD FACSAria cytometer (BD Biosciences) with compensation set based on the staining of each individual fluorochrome alone and correction for autofluorescence with unstained cells. Controls included cells stained with directly conjugated isotype control Abs (BD Biosciences) to assess the degree of nonspecific staining. Results are presented as the percentage of viable neutrophils, macrophages, and microglia recovered from MyD88 KO animals compared to WT mice (set to 100%).

After sorting, viable neutrophils, macrophages, and microglia recovered from *C. koseri *infected MyD88 WT and KO mice were enumerated and added to 96-well plates without further stimulation for 24 h, whereupon conditioned medium was collected to quantitate proinflammatory mediator expression using Milli-Plex multi-analyte bead arrays. Results are expressed as the concentration of each inflammatory mediator per 10^3 ^cells for normalization.

### Processing of brain tissues for histological and immunofluorescence analysis

Brain tissues were collected from *C. koseri *infected MyD88 KO and WT animals at 24 h post-injection to evaluate neuropathological changes. To prepare tissues for immunostaining, mice were perfused with 4% paraformaldehyde (PFA) in 1X PBS using a peristaltic perfusion pump at a flow rate of 10 ml/min. Brain tissues were post-fixed in cold 4% PFA for 1 h and cryoprotected in 30% sucrose prior to freezing and embedding in OCT (Optimal Cutting Temperature compound, Sakura Finetek USA Inc., Torrance, CA) for cryostat sectioning. Serial sections of brain tissues (10 μm) were prepared and processed for immunofluorescence staining with Iba-1 (Biocare Medical, Concord, CA) to assess microglial/macrophage reactivity and imaged using a Zeiss LSM 510 META confocal microscope (Zeiss, New York, NY). In addition, infected brain tissues were processed for Gram staining to visualize extracellular *C. koseri *and the extent of bacterial dissemination.

### Purification of primary astrocytes by FACS

Primary astrocytes were derived from mixed glial cultures of TLR4 WT, TLR4 mutant, C57BL/6 WT, and MyD88 KO mice (1-4 days of age) as previously described [24]. One concern when working with primary astrocytes relates to the issue of cell purity, principally due to low levels of residual microglia [25]. Therefore, after a minimum of three passages while cultured in the presence of 0.1 mM L-LME, a microglial cytotoxic agent [25,26], the resulting cells were stained with CD11b, a cell surface marker expressed on microglia, but not astrocytes, and collected by negative selection to obtain highly enriched astrocytes. Briefly, cells were incubated with Fc Block™ followed by a CD11b-PerCP-Cy5.5 antibody (both from BD Biosciences). Cells were sorted on a FACSAria (BD Biosciences) at a low flow rate through a large nozzle to minimize astrocyte damage during the sorting procedure. Post-sort analysis revealed less than 1% contamination with CD11b^+ ^microglia. An additional assessment of astrocyte purity after sorting was obtained by culturing astrocytes on glass cover slips followed by immunofluorescence staining with GFAP and CD11b (Additional File 1, Figure S2).

### Enzyme linked immunosorbent assay (ELISA)

Quantitation of CCL2 and TNF-α (mouse OptEIA, BD Biosciences) and CXCL2 and IL-1β (mouse DuoSet; R & D Systems, Minneapolis, MN) levels in conditioned supernatants from astrocytes treated with *C. koseri *was performed using standard sandwich ELISA kits according to the manufacturer's instructions.

### Nitrite assay

Levels of nitrite, a stable end product of nitric oxide (NO) following its reaction with O_2_, were quantitated in conditioned supernatants of astrocytes treated with *C. koseri *using the Griess reagent (0.1% naphtyletylenediamine dihydrochloride, 1% sulfanilamide, and 2.5% phosphoric acid, all from Sigma). The absorbance at 550 nm was measured on a plate reader (Spectra Max 190; Molecular Devices, Sunnyvale, CA, USA), and nitrite concentrations determined using a standard curve with sodium nitrite (NaNO_2_; Sigma; level of sensitivity 0.4 μM).

### Statistical analysis

Significant differences were determined either by a Student's *t*-test or one-way analysis of variance (ANOVA) followed by the Holm-Sidak method for multiple pair-wise comparisons as indicated (SPSS Science, Chicago, IL). For all analyses, a *p*-value of less than 0.05 was considered to be statistically significant.

## Results

### MyD88-dependent signals are critical for bacterial containment and inflammatory mediator production during *C. koseri *brain infection

To evaluate the functional importance of MyD88-dependent pathways for CNS immune responses to *C. koseri*, we employed a mouse model of meningitis and brain abscess formation using a *C. koseri *clinical isolate [20]. After subjecting C57BL/6 mice to a series of escalating inoculums, a dose of approximately 3 × 10^4 ^cfu resulted in a 30% mortality rate concomitant with meningitis and ventriculitis, and brain abscess formation in a subset of animals (Additional File 2, Figure S1). Therefore, this dose was used for all subsequent experiments.

**Figure 1:**
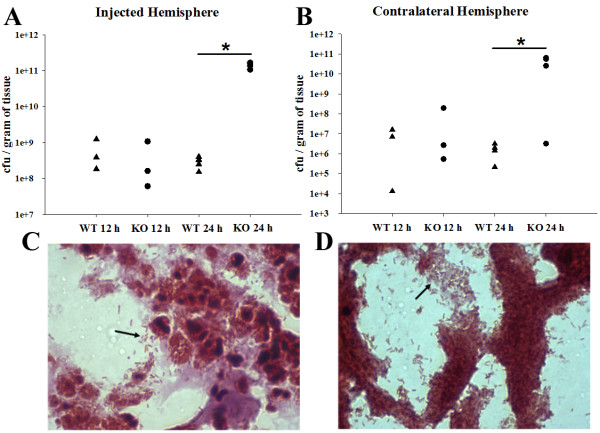
**MyD88-dependent signals are essential for *C. koseri *containment during CNS infection**. MyD88 WT and KO mice (triangles and circles, respectively; 3-4 per group) received intracerebral injections of *C. koseri *(3.4 × 10^4 ^cfu), whereupon the number of viable bacteria in either the injected (A) or contralateral (B) hemispheres were determined at 12 or 24 h post-infection. Bacterial burdens are expressed the colony forming units (cfu) of *C. koseri *per gram of wet tissue weight (each data point represents an individual animal) and significant differences between MyD88 KO and WT mice are denoted by asterisks (*, *t-*test, *p *< 0.05). Identification of *C. koseri *localization by Gram staining revealed heightened numbers of extracellular bacteria (arrows, 100×) associated with the infected hemispheres of MyD88 KO (D) compared to WT (C) animals at 24 h post-infection. Results are representative of three independent experiments with a total of 3-6 mice/group/study.

MyD88 KO mice were exquisitely sensitive to intracerebral *C. koseri*, with the majority of animals succumbing to infection within 24-36 h following bacterial exposure (data not shown). Because MyD88 was essential for survival, this dictated acute sampling intervals of 12 and 24 h following bacterial exposure to avoid potential artifacts due to "survival bias". Bacterial burdens were similar between MyD88 KO and WT mice at 12 h post-infection (Figure 1A and 1B). In contrast, *C. koseri *titers were significantly elevated in both the injected and contralateral hemispheres of MyD88 KO mice at 24 h following bacterial exposure compared to WT animals (Figure 1A and 1B), revealing a pivotal role for MyD88 in controlling *C. koseri *infection. This finding was corroborated by Gram-staining, where MyD88 KO mice exhibited greater extracellular *C. koseri *compared to WT animals (Figure 1D and 1C, respectively). Collectively, these findings reveal the critical role that MyD88-dependent signals play in bacterial containment during CNS *C. koseri *infection.

**Figure 2:**
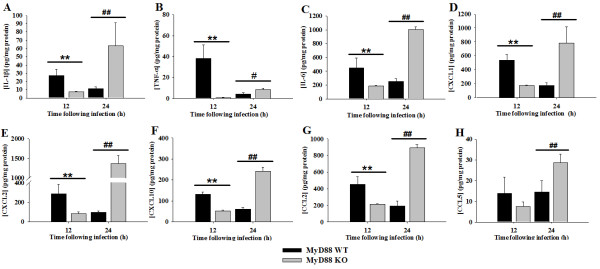
**Proinflammatory mediator production following CNS *C. koseri *infection is influenced by both MyD88-dependent and -independent signals**. MyD88 WT and KO mice (4-6 per group) received intracerebral injections of *C. koseri *(3.4 × 10^4 ^cfu), whereupon supernatants were prepared from infected hemispheres and cytokine/chemokine expression evaluated using multiplex microbead arrays for IL-1β (A), TNF-α (B), IL-6 (C), CXCL1 (D), CXCL2 (E), CXCL10 (F), CCL2 (G), and CCL5 (H). Cytokine/chemokine expression was normalized based on the amount of total protein obtained for each sample to correct for differences in tissue sampling size. Significant differences in mediator expression between MyD88 WT and KO mice at 12 h post-infection are denoted by asterisks (*, *p *< 0.05, **, *p *< 0.01, *t*-test), whereas significant differences in mediator levels between MyD88 WT and KO animals at 24 h post-infection are denoted by hatched signs (#, *p *< 0.05, ##, *p *< 0.01, *t*-test). Results are representative of two independent experiments with a total of 3-6 mice/group/study.

The dramatic sensitivity of MyD88 KO mice to intracerebral *C. koseri *suggested defects in mounting a protective antibacterial inflammatory response in the CNS parenchyma. To examine this possibility, proinflammatory mediator expression was evaluated in MyD88 KO and WT animals using a multi-analyte bead array. Indeed, the expression of numerous cytokines (i.e. IL-1β, TNF-α, and IL-6) and chemokines (i.e. CXCL1, CXCL2, CXCL10, and CCL2) were significantly attenuated in infected MyD88 KO mice at 12 h following bacterial exposure, reflecting impaired immune activation (Figure 2). Surprisingly, the levels of these same mediators were significantly increased in MyD88 KO animals at 24 h post-infection, suggesting a late induction of MyD88-independent signaling to elicit proinflammatory mediator release (Figure 2). It is possible that this MyD88-independent response resulted from heightened bacterial burdens observed in MyD88 KO animals at this interval post-infection (Figure 1).

### Neutrophil and macrophage infiltration and activation into *C. koseri*-infected brain parenchyma is attenuated in MyD88 KO mice

**Figure 3:**
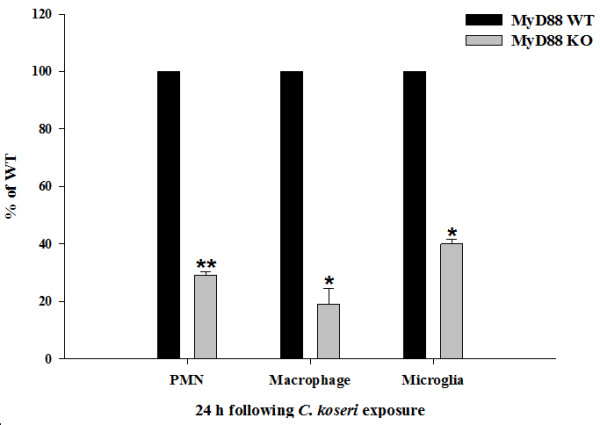
**Neutrophil and macrophage influx into *C. koseri-*infected brain is dictated by MyD88-dependent signaling**. MyD88 KO and WT mice (5-8 per group) received intracerebral injections of *C. koseri *and were sacrificed at 24 h following bacterial exposure, whereupon the percentages of viable neutrophils (Ly-6G^+^, F4/80^-^, and CD45^high^), macrophages (Ly-6G^-^, F4/80^+^, and CD45^high^), and microglia (Ly-6G^-^, F4/80^+^, and CD45^low-intermediate^) were quantitated by FACS analysis with the vital stain 7-AAD. Results are expressed as the percentage of cells normalized to WT values (set to 100%) and represent the mean ± SEM from three independent experiments. Significant differences between MyD88 KO versus WT mice are denoted by asterisks (*, *p *< 0.05, **, *p *< 0.01, *t*-test).

**Figure 4:**
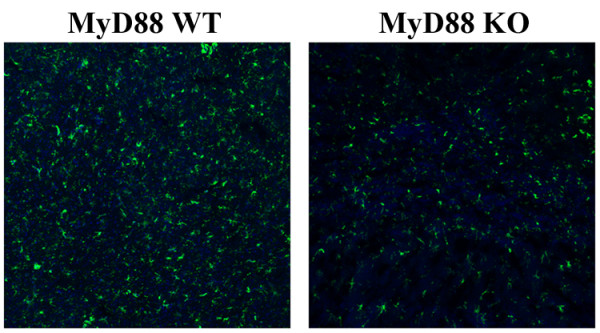
**Microglial/macrophage activation in response to *C. koseri *infection is impaired in MyD88 KO mice**. MyD88 KO and WT mice (6 per group) were sacrificed at 24 h following *C. koseri *exposure, whereupon brain tissues were fixed by paraformaldehyde perfusion and processed for cryostat sectioning. Ten μm thick brain sections were subjected to immunofluorescence staining using Iba-1 (green) and imaged by confocal microscopy (magnification, 10×). Results are representative of six independent animals per group.

The differential impact of MyD88 signaling on chemokine expression at 12 versus 24 h post-infection led us to examine whether these changes correlated with alterations in peripheral immune cell influx. To quantitate the potential differences in neutrophils, macrophages, and microglia between MyD88 KO and WT mice, three-color FACS analysis was utilized. A dramatic reduction in neutrophil and macrophage infiltrates was observed in MyD88 KO mice at 12 h following *C. koseri *exposure (data not shown), which correlated with the diminished chemokine expression observed at this time point (Figure 2). Interestingly, neutrophil and macrophage influx remained significantly lower in MyD88 KO compared to WT animals at 24 h post-infection (Figure 3), which contrasted with the heightened chemokine expression observed at this interval (Figure 2). The percentage of microglia recovered following *C. koseri *infection was also significantly attenuated in MyD88 KO mice, which may be a consequence of the extensive necrosis that accompanied infection in these animals. In addition, Iba-1 staining revealed that macrophage/microglial reactivity was attenuated in MyD88 KO animals (Figure 4), which corroborates the decreased percentages of each as revealed by FACS.

**Figure 5:**
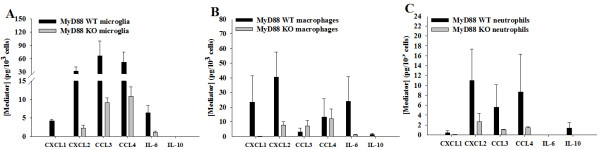
**MyD88 signaling regulates inflammatory mediator production by microglia and infiltrating immune cells associated with CNS *C. koseri *infection**. Viable microglia (A), macrophages (B), and neutrophils (C) were recovered from lesions of MyD88 KO and WT mice at 24 h following *C. koseri *infection by FACS based on exclusion of the vital dye 7-AAD. Cells were cultured overnight without additional stimulation and conditioned supernatants were analyzed for mediator expression by multi-analyte microbead arrays. The amount of each cytokine/chemokine was normalized based on total cell numbers and represents the mean ± SEM from three independent experiments.

To determine whether viable abscess-associated neutrophils, macrophages, or microglia recovered from the infected CNS of MyD88 KO or WT mice displayed any differences in inflammatory mediator expression, we performed microbead array analysis on conditioned supernatants from each cell type following a 24 h incubation period *in vitro *without bacterial re-stimulation, in an attempt to capture cellular activation states that were ongoing *in vivo*. MyD88 was important for eliciting microglial proinflammatory mediator release during CNS *C. koseri *infection as demonstrated by decreased production of CXCL1, CXCL2, CCL3, CCL4, and IL-6 (Figure 5A). This finding agrees with our previous report where MyD88-dependent signaling was shown to be important for microglial activation in response to *C. koseri in vitro *[27]. Similarly, macrophage activation in response to CNS *C. koseri *infection was also influenced by MyD88 as revealed by attenuated CXCL1, CXCL2, and IL-6 production (Figure 5B). Infiltrating neutrophils associated with *C. koseri *infected brain also required MyD88-dependent activation signals for proinflammatory mediator release (Figure 5C). None of these differences in cytokine/chemokine expression between cell types recovered from MyD88 KO versus WT mice reached statistical significance, although trends towards reduced mediator levels were observed in all three populations. The failure to detect significant changes likely stemmed from the fact that low cell yields were obtained across experiments as well as sizable deviations that cannot be avoided given biological variability. Collectively, these results demonstrate that MyD88-dependent signals are not only important for immune cell recruitment but also their subsequent activation in response to CNS *C. koseri *infection.

### Regulation of bacterial burdens and inflammatory mediator expression during CNS *C. koseri *infection are TLR4-independent

**Figure 6:**
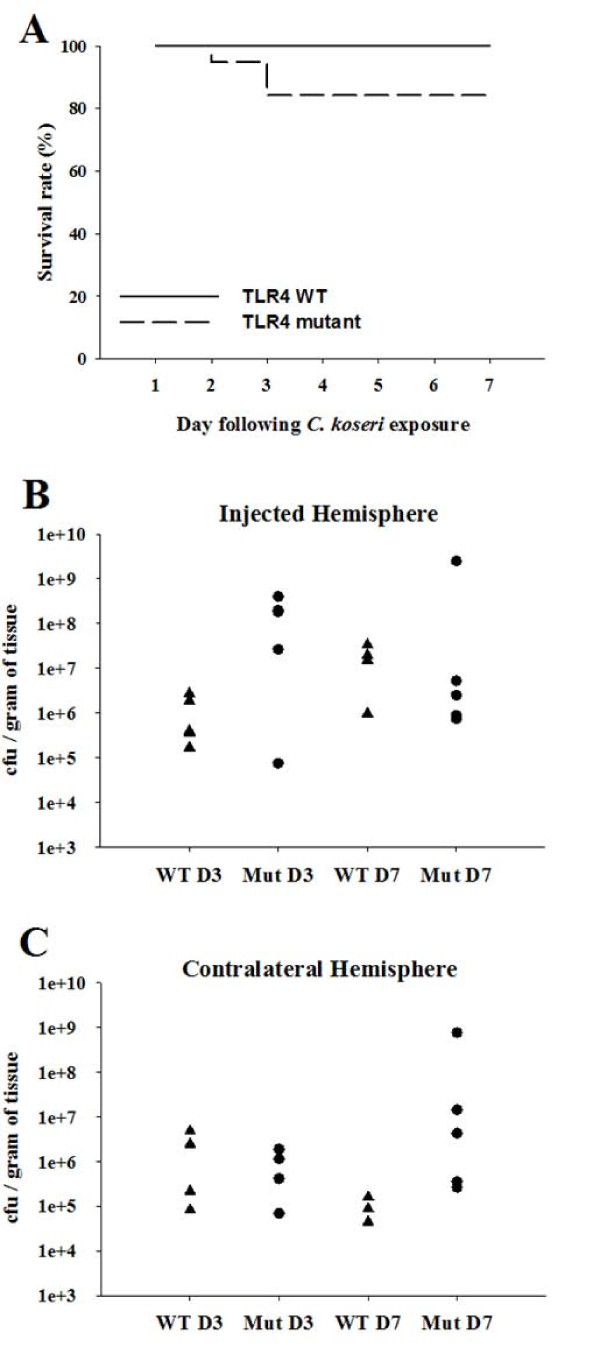
**TLR4 has minimal impact on the course of CNS *C. koseri *infection**. TLR4 WT and TLR4 mutant mice (triangles and circles, respectively; 4-5 per group) received intracerebral injections of *C. koseri *(2.8 × 10^4 ^cfu) to induce meningitis and brain abscess formation. Survival rates of WT and TLR4 mutant mice were 100% and 84%, respectively (A). TLR4 WT or TLR4 mutant (Mut) mice were euthanized at days 3 or 7 post injection (4-5 mice per group), whereupon the number of viable bacteria in either the injected (B) or contralateral (C) hemispheres were determined. Bacterial burdens are expressed as colony forming units (cfu) of *C. koseri *per gram of wet tissue weight (each data point represents an individual animal). Results are representative of three independent experiments with a total of 4-6 mice/group/study.

Since TLR4 has been identified as a key sensor for host defense in several models of Gram-negative infection [28,29] and we observed a dramatic phenotype following *C. koseri *infection in MyD88 KO mice, the contribution of TLR4-mediated signal(s) was next examined. Compared to WT mice, TLR4 mutant animals exhibited slightly lower survival rates following *C. koseri *infection (Figure 6A). The differences in mortality rates observed for MyD88 and TLR4 WT animals can best be explained by the different genetic backgrounds of the two strains (C57BL/6 and C3H, respectively). Indeed, there are several examples in the literature where differences in strain sensitivity to pathogens have been demonstrated, most attributable to variations in MHC haplotype [30-33]. Although there was a tendency towards elevated bacterial titers in the ipsilateral and contralateral hemispheres of TLR4 mutant mice at days 3 and 7 post-infection (Figure 6B and 6C, respectively), these differences did not reach statistical significance in a total of five independent experiments (data not shown).

**Figure 7:**
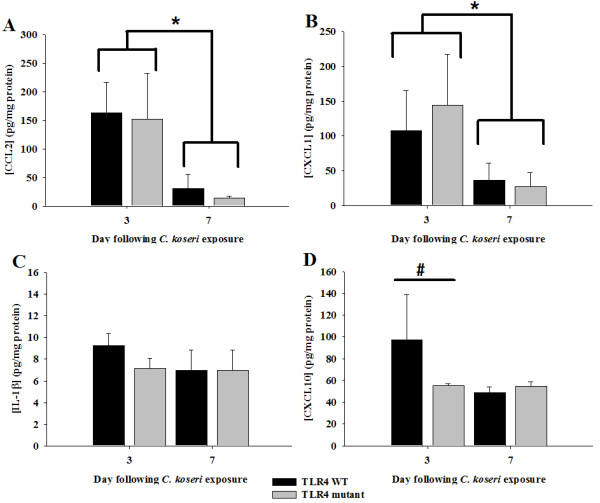
**Proinflammatory mediator production in response to CNS *C. koseri *infection is primarily TLR4-independent**. TLR4 WT and TLR4 mutant mice (4-5 per group) received intracerebral injections of *C. koseri *(2.8 × 10^4 ^cfu), whereupon supernatants were prepared from infected hemispheres and cytokine/chemokine expression evaluated using multiplex microbead arrays for CCL2 (A), CXCL1 (B), IL-1β (C), and CXCL10 (D). Cytokine/chemokine expression was normalized based on the amount of total protein obtained for each sample to correct for differences in tissue sampling size. Significant differences in mediator expression between days 3 and 7 post-infection are denoted by asterisks (*, *p *< 0.05, One-Way ANOVA), whereas the difference in CXCL10 levels between TLR4 WT mice and TLR4 mutant mice at day 3 post-infection is denoted by a hatched sign (#, *p *< 0.05, One-Way ANOVA). Results are representative of three independent experiments with a total of 4-6 mice/group/study.

To evaluate whether TLR4 signaling impacted the inflammatory milieu following *C. koseri *infection, proinflammatory mediator expression was quantified using a microbead array. In general, chemokine expression was significantly higher at day 3 compared to day 7 post-infection, regardless of mouse strain (Figure 7A and 7B). Only CXCL10 expression was significantly attenuated in TLR4 mutant mice at day 3 post-infection (Figure 7D), whereas the majority of mediators, including CCL2, CXCL1, and IL-1β were similar between TLR4 mutant and WT mice across all time points examined (Figure 7). The similarity in proinflammatory mediator expression between WT and TLR4 mutant mice are in agreement with the nearly equivalent bacterial burdens (Figure 6B and 6C). Collectively, these findings indicate that TLR4-mediated signaling does not play a major role in controlling *C. koseri *infection and the resultant inflammatory response, at least under the conditions examined in the present study.

### MyD88, but not TLR4, is essential for astrocyte chemokine production in response to *C. koseri*

**Figure 8:**
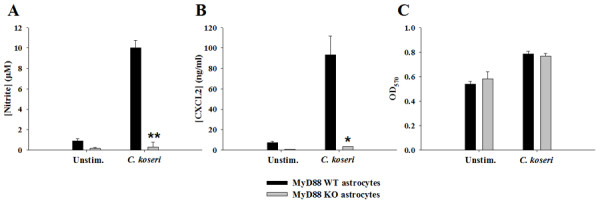
**MyD88-dependent signals play a pivotal role in mediating astrocytic inflammatory mediator release in response to *C. koseri***. Primary astrocytes recovered from MyD88 WT or KO mice were plated at 1 × 10^5 ^cells/well in 96-well plates and exposed to heat-inactivated *C. koseri *(10^7 ^cfu/well) for 24 h. The levels of NO (A) and CXCL2 (B) produced by astrocytes were quantitated by nitrite assay and ELISA, respectively. Astrocyte viability was assessed by a standard MTT assay and the raw OD_570 _absorbance values are reported (C). Significant differences in inflammatory mediator expression between *C. koseri *stimulated MyD88 KO and WT astrocytes are denoted by asterisks (* *p *< 0.05; ** *p *< 0.001, *t*-test). Results are reported as the mean ± SD of three independent wells for each experimental treatment and were identical across three separate experiments.

Astrocytes are capable of bacterial recognition and serve as a major source of chemokines in response to CNS infection/injury [13,14,24]. To date, no studies have demonstrated whether astrocytes are responsive to *C. koseri*, which is relevant since astrocytes are the most numerous cell type in the CNS and would directly encounter bacteria during CNS colonization at the blood-brain barrier as well as within the parenchyma. Only a few reports have examined the role of MyD88-dependent signaling in astrocytes; however, this was in response to purified PAMPs or LCMV [34-36] and not an intact bacterium such as *C. koseri*, which presents cells with a complex milieu of PAMPs. Both NO and CXCL2 production were significantly inhibited in MyD88 KO astrocytes compared to WT cells (Figure 8), indicating that MyD88-dependent pathway(s) are critical for transducing signals required for the production of these inflammatory mediators by astrocytes.

**Figure 9:**
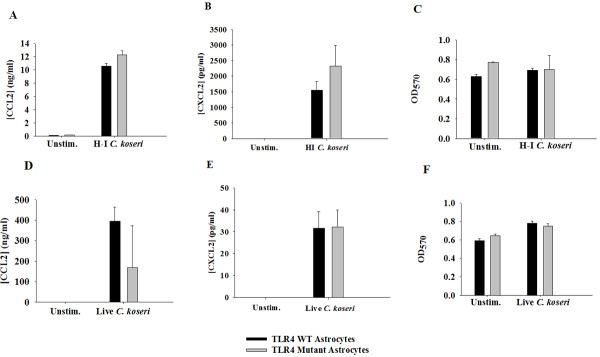
**Astrocyte activation in response to heat-inactivated or viable *C. koseri *is TLR4-independent**. FACS-enriched WT and TLR4 mutant astrocytes were plated at 1 × 10^5 ^cells/well in 96-well plates and exposed to either heat-inactivated *C. koseri *(10^7 ^cfu/well) for 24 h (A, B, C), or live *C. koseri *at a multiplicity of infection (MOI) of 1 for 6 h (D, E, F). CCL2 (A and D) and CXCL2 (B and E) production was quantitated by ELISA. Astrocyte viability was assessed using a MTT assay and the raw OD_570 _absorbance values are reported (C, F). There were no significant differences in chemokine release between WT and TLR4 mutant astrocytes. Results are reported as the mean ± SD of three independent wells for each experimental treatment and were identical across six separate experiments.

To assess the functional importance of TLR4 in inducing astrocytic inflammatory mediator production in response to *C. koseri*, we compared responses of WT and TLR4 mutant astrocytes enriched by FACS analysis to remove residual contaminating microglia because of the controversy regarding TLR4 expression in astrocytes [37-39]. Both CCL2 and CXCL2 production was equivalent between WT and TLR4 mutant astrocytes in response to heat-inactivated *C. koseri *(Figure 9A and 9B, respectively), suggesting *C. koseri *recognition by astrocytes is TLR4-independent. Importantly, IL-1β and TNF-α were below the limit of detection by ELISA (data not shown) indicating that *C. koseri *is not strong inducer of cytokine expression in astrocytes.

As virulence factors produced by viable *C. koseri *may contribute to astrocyte activation via pathways distinct from those elicited by heat-inactivated bacteria, we next studied astrocyte responses to live *C. koseri*. Similarly, astrocyte activation by live *C. koseri *was also TLR4-independent (Figure 9D and 9E). Neither heat-inactivated nor live *C. koseri *led to significant changes in astrocyte viability as revealed by MTT assays (Figure 9C and 9F, respectively). Taken together, these data suggest that enriched astrocytes recognize *C. koseri *and respond with robust production of chemokines in a MyD88-dependent, TLR4-independent manner.

## Discussion

*C. koseri *is a Gram-negative pathogen that can cause meningitis and ventriculitis, which frequently invades the surrounding brain parenchyma resulting in multiple abscesses [1,2]. Although a few reports have examined various aspects of *C. koseri *meningitis/brain abscess in rodent models [40-42], little is known regarding the mechanism(s) responsible for bacterial recognition and inflammatory responses within the CNS. In addition, the impact of TLR signaling in CNS Gram-negative infections has received less attention compared to the number of studies focusing on Gram-positive pathogens [7,8,21]. This is an important point since recent evidence indicates that cytokine responses are tailored for specific pathogen classes and are not relegated to a "one size fits all" response [3-5]. Indeed, this point is highlighted by comparing the current study with an earlier report from our laboratory where CNS infection with *S. aureus *was examined. Both utilized MyD88 KO mice; however, here we demonstrate that *C. koseri *was capable of eliciting cytokine/chemokine production via MyD88-independent pathways, whereas *S. aureus *recognition was strictly MyD88-dependent [6]. Another distinction was that MyD88 was essential for *C. koseri *containment, whereas this was not the case with *S. aureus *where bacterial titers were similar between MyD88 KO and WT mice [6]. Similar differences between MyD88 involvement in CNS *C. koseri *versus *S. aureus *infection were observed in a follow up study from our group. Namely, MyD88-dependent signaling was critical in CNS intrinsic cells to generate maximal innate immune responses during CNS *S. aureus *infection, whereas little impact of MyD88-independent pathways was observed [43]. However, we did find that astrocytic chemokine induction following *C. koseri *stimulation was MyD88-dependent, suggesting some degree of conservation between pathogen sensing mechanisms. Collectively, these findings highlight the ability of CNS immune mechanisms to discriminate between Gram-positive versus -negative species. The level of sophistication for pathogen recognition (i.e. whether distinctions can be made within similar pathogen classes) remains to be determined.

Our results revealed an intriguing dichotomy for MyD88-dependent signals in regulating host immunity during CNS *C. koseri *infection. Specifically, MyD88 was pivotal for inducing proinflammatory mediator release immediately following bacterial exposure (i.e. 12 h); however, within 24 h a transition occurred to elicit these same mediators via a MyD88-independent mechanism, with cytokine/chemokine levels in MyD88 KO mice exceeding those detected in infected WT animals. This deferred induction of MyD88-independent effects is in agreement with the delayed kinetics of this signaling pathway as reported by others [44,45]. However, it was apparent that the elevated MyD88-independent proinflammatory response elicited in KO mice at 24 h was unable to impact *C. koseri *clearance since bacterial burdens were significantly elevated in MyD88 KO compared to WT mice, reaching approximately 3-log higher levels in the former. However, another interpretation is that MyD88-mediated responses increase anti-inflammatory mediator expression that would normally repress potentially damaging inflammation while promoting the production of anti-microbial substances. In this case, MyD88 loss would be expected to attenuate anti-inflammatory mediator release, allowing the MyD88-independent production of damaging molecules to remain unchecked. However, we did not observe any significant differences in IL-10 expression in MyD88 KO or WT mice at either time point following *C. koseri *infection (data not shown). Nonetheless, we cannot exclude the possibility that alternative immune suppressive mediators not examined here (i.e. TGF-β, suppressor of cytokine signaling (SOCS) proteins) are driven by MyD88-dependent signaling, and the absence of these molecules exacerbates late stage *C. koseri *inflammatory responses. With regard to the mechanism(s) responsible for eliciting MyD88-independent inflammation during *C. koseri *infection, one possibility is signaling via the alternative adaptor molecule TRIF. Indeed, activation of TRIF-dependent signaling leads to the expression of IFN-inducible genes and late phase NF-κB activation [17,46]. However, the only TLRs that utilize TRIF are TLR3 and TLR4 and the former is likely not involved in *C. koseri *recognition since its ligand, dsRNA, is typical of viral infections [47]. Likewise, our results demonstrate a minor role for TLR4 in terms of eliciting proinflammatory mediator release and bacterial containment. Therefore, the contribution of TRIF towards the MyD88-independent proinflammatory response during *C. koseri *CNS infection remains uncertain. Other potential candidates include the cytoplasmic PRRs NOD1 and 2 that sense PGN moieties as well as NLRs that have been implicated in the recognition of flagellated bacteria such as Ipaf and Naip [48-50]. However, the involvement of these alternative PRRs in regulating proinflammatory mediator release during CNS *C. koseri *infection waits testing in future studies.

One unexpected finding was that elevated chemokine expression in MyD88 KO animals at 24 h post-infection did not translate into enhanced immune cell recruitment. Rather, neutrophil and macrophage infiltrates remained significantly attenuated in MyD88 KO mice compared to WT animals. The reason for this finding is unclear; however, since multiple events are required for immune cell extravasation across the blood-brain barrier, it is possible that another phase is aberrant in MyD88 KO mice. For example, early IL-1β and TNF-α production is important for inducing adhesion molecule expression on BBB endothelial cells [51-53]. Since the production of both cytokines is significantly blunted early in MyD88 KO animals, sufficient time would be required to induce adhesion molecule expression to overcome the migration block. In this case, by the time that IL-1β and TNF-α were upregulated in MyD88 KO animals, this may prove too late to impact immune cell migration into the CNS. Yet, even those neutrophils and macrophages that were recruited to the site of infection in MyD88 KO mice were less activated as revealed by reduced levels of cytokine/chemokine production. It is possible that the residual recruitment of these leukocyte subsets in MyD88 KO animals resulted from alternative chemotactic factors such as microbial-derived peptides (i.e. formyl peptides) or complement split products (i.e. C3a or C5a). However, once these cells enter the CNS parenchyma they are unable to effectively respond to *C. koseri *due to the lack of MyD88 signaling. Since heightened inflammatory mediator release was evident in brain abscess homogenates but not purified immune cell populations at 24 h post-infection, this suggests MyD88-independent contributions from alternative cell types, the identity of which remains unknown at the present time.

The host possesses multiple redundant mechanisms to ensure rapid immune responses to pathogens and infection resolution [9,10,48,54]. When taken together, our results strongly suggest a collaborative role for numerous recognition pathways in generating a potent anti-bacterial immune response to CNS *C. koseri *infection. First, since the majority of TLRs utilize MyD88 as an adaptor, MyD88 deficiency will globally abolish all TLR-mediated signals in addition to signaling via the IL-1R and IL-18R. This is particularly relevant since *C. koseri *harbors numerous ligands that can stimulate MyD88-dependent TLR signaling including lipoproteins (TLR2), LPS (TLR4), flagella (TLR5), and CpG DNA (TLR9) as well as the ability of *C. koseri *to elicit IL-1β release during CNS infection (Figure 2). In addition to TLRs, cytoplasmic PRRs such as NLRTC4 and NAIP that are activated by flagellin [48] could also contribute to proinflammatory mediator production since *C. koseri *is a flagellated bacterium; however, this possibility remains speculative.

The concept of receptor redundancy for eliciting maximal responses to *C. koseri *was also evident when examining primary astrocytes isolated from MyD88 KO or TLR4 mutant mice. Similar to our *in vivo *observations, chemokine production was negligible in MyD88 KO astrocytes in response to intact bacteria, whereas TLR4 mutant cells responded identically to their WT counterparts. This indicates that MyD88-dependent receptors are critical for astrocyte activation and potential candidates include TLR2, TLR5, and TLR9 that recognize lipoproteins, flagellin, and CpG DNA, respectively. Although it is clear that TLR4 is not involved in astrocyte responses to intact *C. koseri*, the extent of TLR4 expression in astrocytes and whether it plays a role in LPS sensing remains controversial [37-39]. This issue has been hampered by the poor quality of commercially available TLR4 antibodies in the mouse as well as concerns regarding the purity of primary astrocyte cultures [25]. However, as this topic was not the focus of the current report, future studies using highly purified astrocytes obtained by FACS analysis or alternative methods such as positive selection via magnetic beads are needed to address these issues related to LPS responsiveness.

In summary, MyD88 is crucial for eliciting a protective host immune response during *C. koseri *brain infection. Despite a late-phase induction of MyD88-independent signaling to trigger proinflammatory release, this response was not sufficient to negate uncontrolled bacterial replication and lethality in MyD88 KO animals. The fact that TLR4 had little impact on the course of infection strongly suggests the involvement of alternative TLRs as well as the IL-1R and IL-18R, which also utilize MyD88 as an adaptor. The identity of these alternative MyD88-dependent receptors remains the topic for future studies.

## List of Abbreviations

7-AAD: 7-Aminoactinomycin D; CFU: colony forming unit; DMSO: dimethyl sulfoxide; FACS: fluorescent activated cell sorting; HBSS: Hank's Balanced Salt Solution; IFN: interferon; IRF3: interferon regulatory factor 3; KO: knockout; LPS: lipopolysaccharide; MyD88: myeloid differentiation factor 88; NLR: NOD-like receptor; NOD1: nucleotide-binding oligomerization domain containing 1; PAMP: pathogen-associated molecular pattern; PRR: pattern recognition receptor; TLR: Toll-like receptor; TRAM: TRIF-related adaptor molecule; TRIF: TIR-domain-containing adaptor protein-inducing IFN-β; WT: wild type.

## Competing interests

The authors declare that they have no competing interests.

## Authors' contributions

SL performed the experiments, participated in study design, and helped to draft the manuscript. TK conceived the study, participated in study design, and wrote the manuscript. Both authors have read and approved the final version of the manuscript.

## Supplementary Material

Additional file 1 **Astrocyte enrichment by sub-culturing and FACS**. Primary astrocytes were sub-cultured by shaking and passage three times before collection. Astrocytes were harvested by trypsinization and stained with a CD11b antibody conjugated to PerCP-Cy5.5. The majority of cells recovered from astrocyte flasks were CD11b-negative, with an average of 3-10% contaminating CD11b-positive microglia (A). Residual CD11b-positive cells were depleted from astrocytes by sorting, as indicated by post-sort analysis (B). Purified microglia were included as a positive control for CD11b staining (C). Subsequently, sorted astrocytes were plated on cover slips at a density of 1 × 10^4 ^cells/ml. After 24 h, cells were stained for GFAP (D) and Iba-1 (E) to visualize astrocytes and residual microglia, respectively. Purified microglia were stained with Iba-1 as positive control (F). Click here for file

Additional file 2 **Establishment of CNS *C. koseri *infection and brain abscess formation**. (A) C57BL/6 mice (4-5 per group) were used to optimize *C. koseri *infectious doses and survival rates out to day 7 post-infection are presented. (B) A brain abscess induced by *C. koseri *at day 7 post-infection is shown (arrows; magnification, 12.5×). Click here for file
