# Supplementary figures and images for: MyD88 is pivotal for immune recognition of Citrobacter koseri and astrocyte activation during CNS infection†
Source: J Neuroinflammation. 2011 Apr 16;8:35. doi: 10.1186/1742-2094-8-35 (PMC3101120; doi:10.1186/1742-2094-8-35)

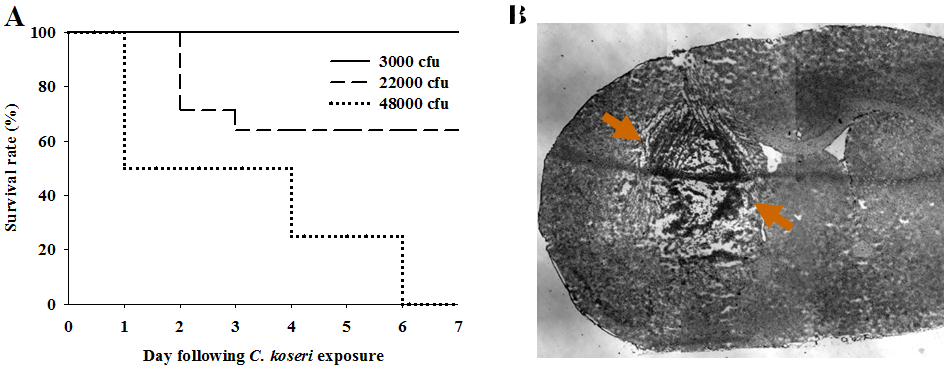

Supplement: Additional file 1 — Astrocyte enrichment by sub-culturing and FACS. Primary astrocytes were sub-cultured by shaking and passage three times before collection. Astrocytes were harvested by trypsinization and stained with a CD11b antibody conjugated to PerCP-Cy5.5. The majority of cells recovered from astrocyte flasks were CD11b-negative, with an average of 3-10% contaminating CD11b-positive microglia (A). Residual CD11b-positive cells were depleted from astrocytes by sorting, as indicated by post-sort analysis (B). Purified microglia were included as a positive control for CD11b staining (C). Subsequently, sorted astrocytes were plated on cover slips at a density of 1 × 10^4^ cells/ml. After 24 h, cells were stained for GFAP (D) and Iba-1 (E) to visualize astrocytes and residual microglia, respectively. Purified microglia were stained with Iba-1 as positive control (F). [file 1742-2094-8-35-S2.TIFF]

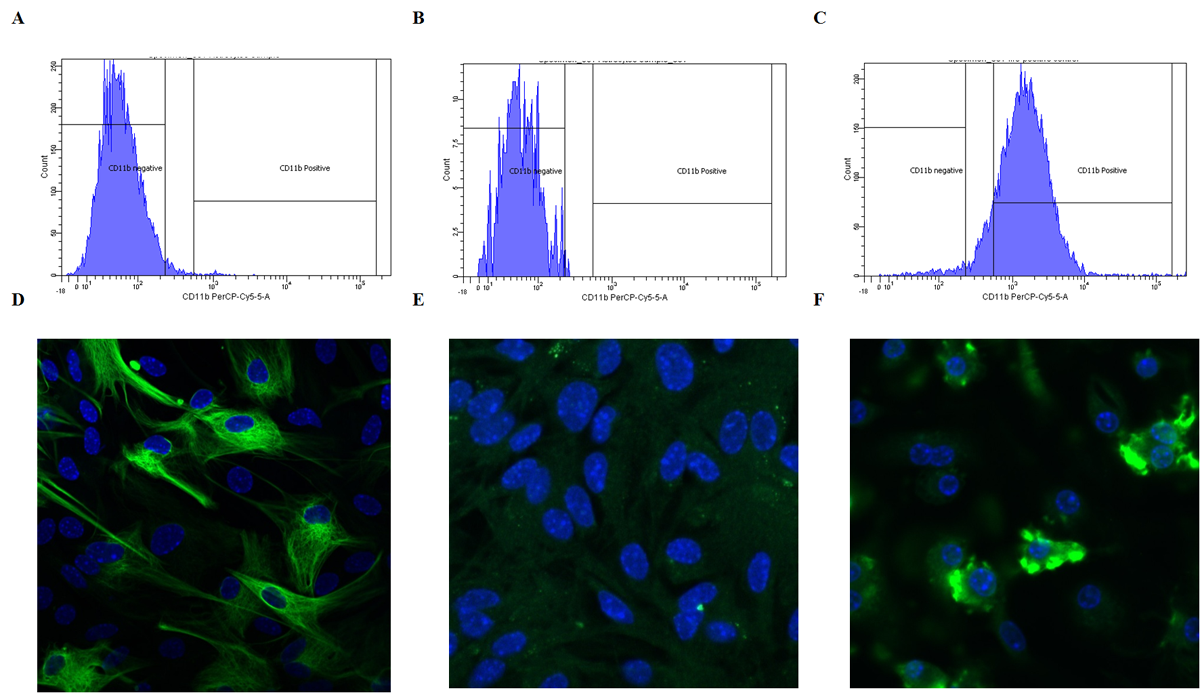

Supplement: Additional file 2 — Establishment of CNS C. koseri infection and brain abscess formation. (A) C57BL/6 mice (4-5 per group) were used to optimize C. koseri infectious doses and survival rates out to day 7 post-infection are presented. (B) A brain abscess induced by C. koseri at day 7 post-infection is shown (arrows; magnification, 12.5×). [file 1742-2094-8-35-S1.TIFF]
